# Effects of equol on multiple K^+^ channels stably expressed in HEK 293 cells

**DOI:** 10.1371/journal.pone.0183708

**Published:** 2017-08-23

**Authors:** Xiu-Ling Deng, Yan Wang, Guo-Sheng Xiao

**Affiliations:** 1 Department of Physiology and Pathophysiology, School of Basic Medical Sciences, Xi’an Jiaotong University Health Science Center, Xi’an, Shaanxi, China; 2 Xiamen Cardiovascular Hospital, Xiamen University, Xiamen, Fujian, China; Georgia State University, UNITED STATES

## Abstract

The present study investigated the effects of equol on cardiovascular K^+^ channel currents. The cardiovascular K^+^ channel currents were determined in HEK 293 cells stably expressing cloned differential cardiovascular K^+^ channels with conventional whole-cell patch voltage-clamp technique. We found that equol inhibited hKv1.5 (IC_50_: 15.3 μM), hKv4.3 (IC_50_: 29.2 μM and 11.9 μM for hKv4.3 peak current and charge area, respectively), *I*_Ks_ (IC_50_: 24.7 μM) and *I*_hERG_ (IC_50_: 31.6 and 56.5 μM for *I*_hERG.tail_ and *I*_hERG.step_, respectively), but not hKir2.1 current, in a concentration-dependent manner. Interestingly, equol increased BK_Ca_ current with an EC_50_ of 0.1 μM. It had no significant effect on guinea pig ventricular action potentials at concentrations of ≤3 μM. These results demonstrate that equol inhibits several cardiac K^+^ currents at relatively high concentrations, whereas it increases BK_Ca_ current at very low concentrations, suggesting that equol is a safe drug candidate for treating patients with cerebral vascular disorders.

## Introduction

Equol [7-hydroxy-3-(4’-hydroxyphenyl)-chroman] is an active metabolite of the soy isoflavone daidzein generated in the intestinal microbial flora in some, but not all, individuals after consuming daidzein[1–3]. Biological activities of equol are greater than daidzein in non-vascular systems[4, 5] with superior antioxidant activity[6–10]. An earlier study from Sobey and colleagues demonstrated that equol dilates carotid artery *in vitro* and basilar artery of normotensive rats *in vivo* with similar potency to its parent compound daidzein. Equol retains its vasorelaxant activity in carotid arteries from hypertensive rats, whereas effects of daidzein were insignificant, suggesting that equol may be a useful therapeutic agent to treat cerebral vascular disorders[11]. A recent study demonstrates that equol producers showed lower serum uric acid, triglyceride, and the ratio of waist and hip, as well as higher HDL-cholesterol, suggesting that equol may reduce cardiovascular risk[12]. Therefore, it is believed that equol rather than daidzein itself may contribute to the beneficial effects of soy foods in preventing cardiovascular disorders.

Our recent study showed that equol increases large-conductance Ca2+-activated K+ (BKCa) current by acting on its auxiliary β1-subunits and contributes to the equol-mediated vasodilation and increase of cerebral blood flow[13]. However, it is unknown whether equol could affect other cardiovascular K+ currents or whether it could prolong ventricular action potentials. The present study was therefore designed to investigate the effects of equol on cloned hKv1.5 (encoding for *I*Kur, ultra-rapidly-delayed rectifier K+ current), hKv4.3 (encoding for cardiac *I*to, transient outward K+ current), recombinant cardiac *I*Ks (hKCNQ1/hKCNE1, slowly-delayed rectifier K+ current), hERG (human Ether-à-go-go-related gene encoding for cardiac *I*Kr, rapidly-delayed rectifier K+ current), stably expressed in HEK 293 cells, and ventricular action potential of guinea pig hearts. Our results demonstrated that equol inhibited these cardiac K+ currents at relatively high concentrations, whereas it increased BKCa current at a low concentration range without prolonging action potential duration in guinea pig ventricular myocytes.

## Materials and methods

### Cell culture

The HEK 293 cell lines stably expressing hKv4.3 (*KCND3*, encoding *I*to)[14], hKv1.5 (*KCNA5*, encoding *I*Kur)[15], hERG (*KCNH2*, encoding *I*Kr)[16], hKCNQ1/hKCNE1 (encoding *I*Ks)[17], Kir2.1 (*KCNJ2*, encoding *I*K1)[18] or KCa1.1/β1 (*KCNMA1/KCNMB1*, encoding BKCa)[19] were generously provided by Dr. Gui-Rong Li, the University of Hong Kong, and maintained in Dulbecco’s modified eagle medium (DMEM, Invitrogen, Carlsbad, CA) supplemented with 10% fetal bovine serum and 400 μg/ml G418 (Sigma-Aldrich, St Louis, MO, USA) for the cell line expressing hKv4.3, hKv1.5, hERG, Kir2.1 and KCa1.1/β1, or 100 μg/ml hygromycin (Invitrogen) for the cell line expressing recombinant cardiac *I*Ks. Cells were seeded on a glass cover slip for electrophysiological recordings.

### Preparation of guinea pig ventricular myocytes

Guinea pigs weighing 250–300 g of either sex were used in the present study. The experimental procedure was approved by the Institutional Ethic Committee of Animal Use for Teaching and Research. After anesthetization of the animal with sodium pentobarbital (40 mg/kg, i.p.), ventricular myocytes were enzymatically dissociated from guinea pig hearts as described previously[20, 21]. The isolated cardiomyocytes were maintained in a high-K+ storage medium at room temperature for 2 h before action potential recording.

### Solution and chemicals

Tyrode’s solution contained (in mM): 140 NaCl, 5.4 KCl, 1 MgCl2, 1.8 CaCl2, 0.33 NaH2PO4, 10 HEPES, 10 glucose, pH was adjusted to 7.4 with NaOH. The pipette solution contained (in mM): 20 KCl, 110 K-aspartate, 1 MgCl2, 10 HEPES, 5 EGTA, and 0.1 GTP, 5 Na2-phosphocreatine, and 5 Mg-ATP (or K2-ATP for *I*Ks or action potential recording), pH was adjusted to 7.2 with KOH. MgCl2 in pipette solution was replaced by NaCl, when recording *I*Ks.

Equol (50% R-equol and 50% S-equol) was obtained from Nanjing Laiyin Medicine Technology Limited Company (Nanjing, Jiangsu, China), and was prepared as 50 mM stock solutions in dimethyl sulfoxide (DMSO, Sigma-Aldrich) and added to the bath solution at the indicated final concentrations. The DMSO concentration in the working solution was <0.2% and did not affect the membrane currents.

### Electrophysiology

Coverslips with HEK 293 cells expressing corresponding ion channels or ventricular myocytes were transferred to an open perfusion chamber (1 ml) mounted on the stage of an inverted microscope (IX50, Olympus, Japan), and superfused at 2–3 ml/min with Tyrode’s solution. The whole-cell patch-clamp technique was used for electrophysiological recording. Borosilicate glass electrodes (1.2-mm OD) were pulled with a Brown-Flaming puller (model P-97, Sutter Instrument Co., Novato, CA, U.S.A.) and had tip resistances of 2–3 MΩ when filled with pipette solution. A 2-M KCl-agar salt bridge was used as reference electrode. The series resistance was compensated by 70–80% to minimize voltage errors. The membrane currents were recorded with an EPC-10 amplifier and Pulse software (HEKA, Lambrecht, Germany). Command pulses were controlled by Pulse software. Current signals were low-pass filtered at 5 kHz. The ionic currents were recorded at room temperature (23–25°C), and cardiac action potentials were recorded at 36–37°C.

### Data analysis

Group data are presented as mean±SEM. Nonlinear curve fitting was performed using Pulsefit (HEKA) and SigmaPlot 12.0 (SPSS Science, Chicago, IL, USA). Paired and/or unpaired Student’s t-tests were used to evaluate the statistical significance of differences between two group means. ANOVA was used for multiple groups. Values of *P*<0.05 were considered statistically significant.

## Results

### Effect of equol on hKv1.5 curren*t*

**Fig 1:**
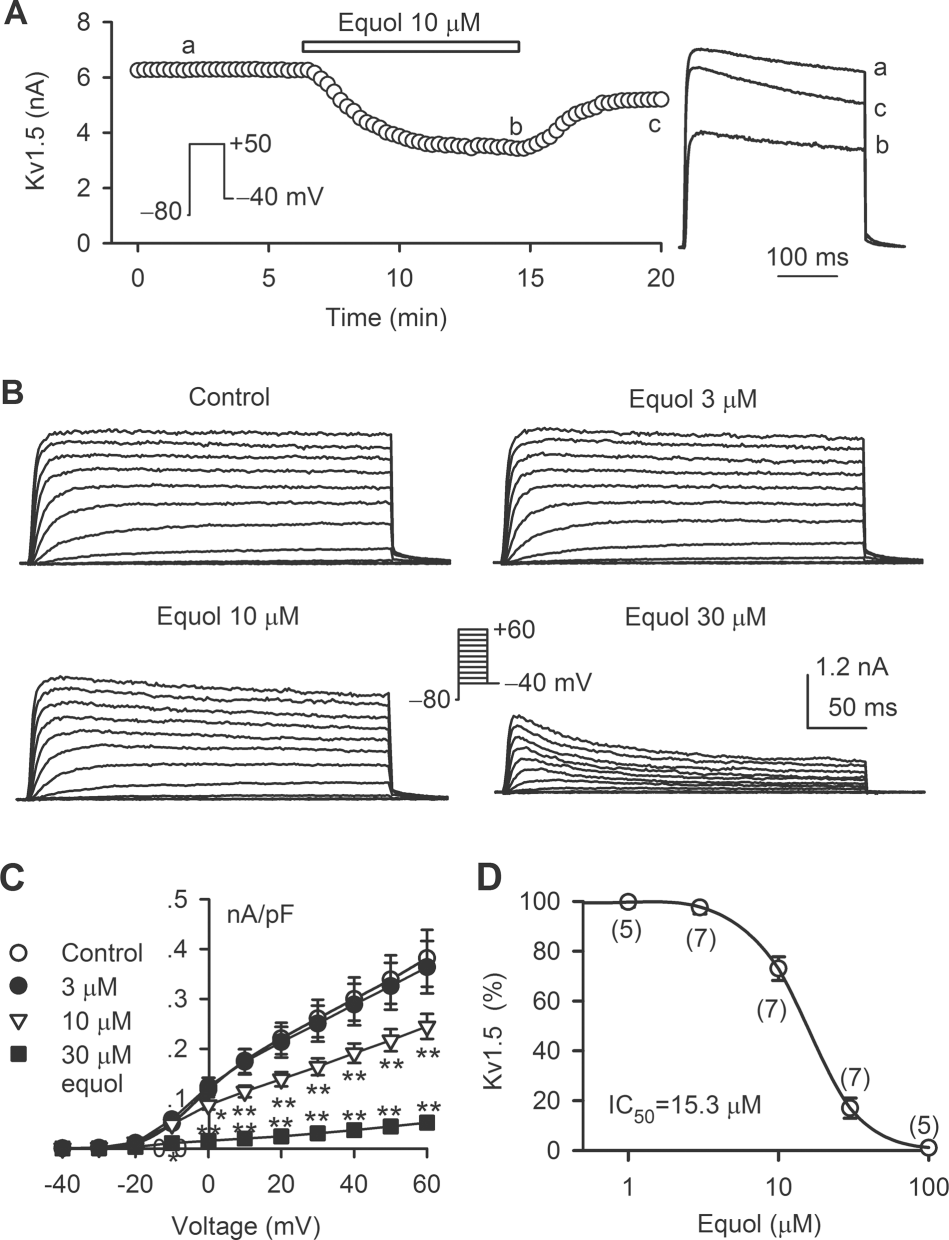
Effect of equol on hKv1.5 current. (A) Time course of hKv1.5 current recorded in a representative HEK 293 cell stably expressing *KCNA5* in the absence and presence of 10 μM equol. Original current traces at corresponding time points are shown in the right. (B) Voltage-dependent hKv1.5 current recorded in a typical experiment in control and in the presence of 3, 10 and 30 μM equol. (C) *I-V* relationships of hKv1.5 current in the absence and presence of 3, 10 and 30 μM equol (n = 7, *P<0.05, **P<0.01 *vs*. control). (D) Concentration-response relationship for inhibiting hKv1.5 current by equol at +50 mV. The data were fitted to the Hill equation: *E* = *E*_max_/[1+(IC_50_/*C*)^b^], where *E* is the inhibition of current in percentage at concentration *C*, *E*_max_ is the maximum inhibition, IC_50_ is the concentration for 50% of maximum inhibition effect, and *b* is the Hill coefficient. The numbers in the parentheses represent experimental number.

Fig 1A shows the time course of the hKv1.5 current recorded in a representative cell in the absence and presence of 10 μM equol with the voltage protocol shown in the inset (300-ms voltage step to +50 mV from −80 mV and then back to −40 mV). Equol gradually inhibited the Kv1.5 current, and the inhibitory effect was partially reversed on washout. Similar results were obtained in 5 other cells. Fig 1B displays the voltage-dependent hKv1.5 current recorded in a typical experiment with the voltage protocol shown in the inset in the absence and presence of 3, 10, and 30 μM equol. Fig 1C illustrates the current-voltage (*I-V*) relationships of hKv1.5 current measured at end of depolarization voltage steps during control and after application of 3, 10 and 30 μM equol. The current was inhibited by 10 and 30 μM equol (n = 7, *P*<0.05 or *P*<0.01 *vs*. control at 0 mV to +60 mV). The concentration-response relationship of equol (1 to 100 μM) for inhibiting hKv1.5 current at +50 mV was fitted to a Hill equation (Fig 1D). The IC_50_ of equol for inhibiting hKv1.5 current was 15.3 μM with a Hill coefficient of 2.3.

### Inhibition of hKv4.3 current by equol

**Fig 2:**
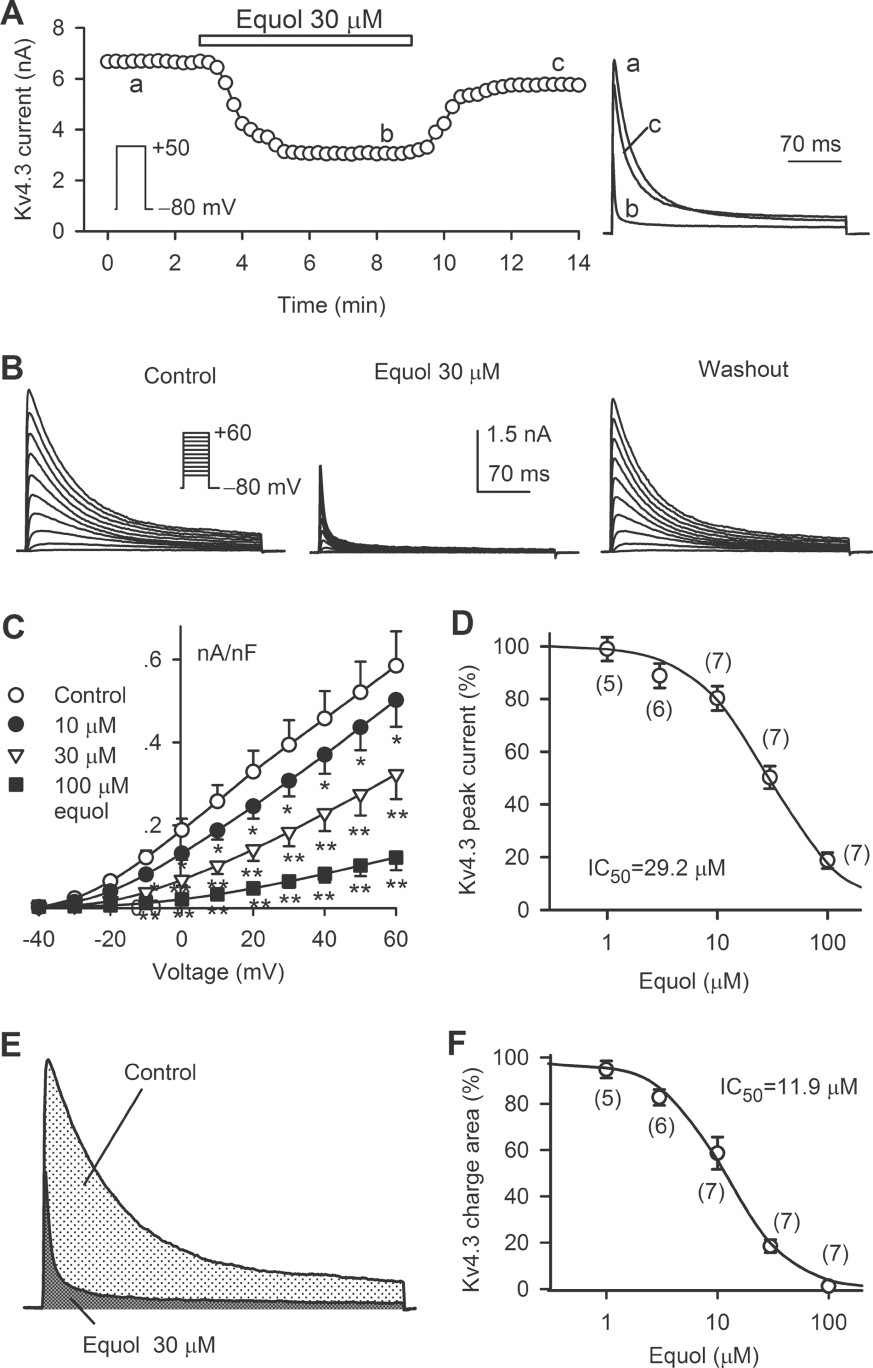
Effect of equol on hKv4.3 current. (A) Time course of hKv4.3 current recorded in a representative HEK 293 cell stably expressing *KCND3* with the voltage protocol shown in the inset in the absence and presence of 30 μM equol. Original current traces at corresponding time points are shown in the right. (B) Voltage-dependent hKv4.3 current recorded in a typical experiment in the absence and presence of 30 μM equol and upon washout. (C) *I-V* relationships of hKv4.3 current in the absence and presence of 10, 30 and 100 μM equol (n = 7, *P<0.05, **P<0.01 *vs*. control). (D) Concentration-response relationship for inhibiting hKv4.3 current by equol at +50 mV. The numbers in the parentheses represent experimental number. (E) Representative hKv4.3 traces for integrating area under current curves in the absence and presence of 30 μM equol. (F) Concentration-response relationship of hKv4.3 charge area reduction by equol at +50 mV was fitted to the Hill coefficient. The numbers in the parentheses represent experimental number.

Fig 2A shows the time course of hKv4.3 current recorded in a HEK 293 cell stably expressing *KCND3* in the absence and presence of 30 μM equol. Equol reversibly inhibited Kv4.3 current. Similar results were obtained in another typical cell for voltage-dependent Kv4.3 current recorded with the voltage protocol shown in the inset (Fig 2B). *I-V* relationships of equol (10, 30, and 100 μM) for inhibiting hKv4.3 current are illustrated in Fig 2C. Equol inhibited hKv4.3 current in a concentration-dependent manner. Significant inhibition of Kv4.3 current was seen at test potentials of −10 mV to +60 mV (n = 7, *P*<0.05 or *P*<0.01 *vs*. control). The IC_50_ of equol for inhibiting hKv4.3 current was 29.2 μM with a Hill coefficient of 1.3 (Fig 2D).

Equol remarkably increased hKv4.3 inactivation (or decay) (Fig 2A and 2B), indicating a typical of open channel blocking effect as described previously[22, 23], and charge area of hKv4.3 current was decreased more than hKv4.3 peak current. We therefore analyzed the charge area[24] of hKv4.3 current at +50 mV by integrating the area under current curve (Fig 2E) with different concentrations of equol. The IC50 of equol for reducing the current charge area was 11.9 μM with a Hill coefficient of 1.5 (Fig 2F).

**Fig 3:**
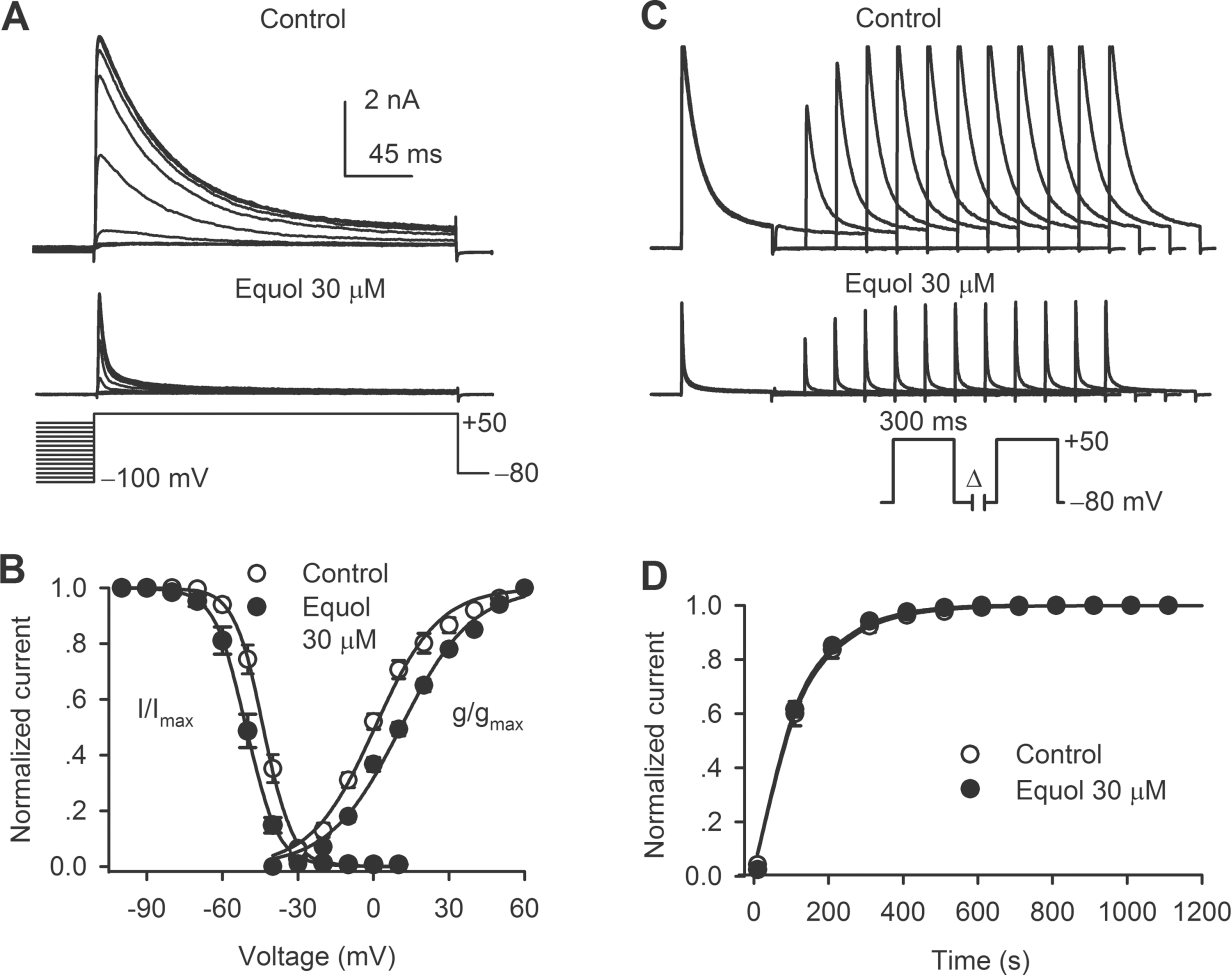
Effect of equol on hKv4.3 kinetics. (A) Voltage protocol and hKv4.3 traces for assessing voltage-dependence of hKv4.3 inactivation in the absence and presence of 30 μM equol. (B) Normalized voltage-dependent variables of hKv4.3 current inactivation (*I/I*_max_, n = 7) and activation (g/g_max_, n = 7) were fitted to the Boltzmann equation. (C) Voltage protocol and current traces for assessing recovery of hKv4.3 from inactivation in the absence and presence of 30 μM equol. (D) Recovery curves of hKv4.3 current from inactivation were fitted to a mono-exponential function.

Fig 3A illustrates the representative current and voltage-protocol used for determining the availability of hKv4.3 current in the absence and presence of 30 μM equol. The variables (Fig 3B) of I/Imax and g/gmax were fitted to a Boltzmann equation: g = 1/{1+exp[(V1/2−Vt)/K]}, where V1/2 is the voltage of 50% channel availability or maximal activation of the channel, Vt is the test potential, and K is slope factor. The V1/2 of hKv4.3 inactivation was −43.8±2.1 mV in control, and was negatively shifted to −50.4±1.9 mV by 30 μM equol (n = 7, *P*<0.01), while the slope factor K was −5.7±1.2 mV in control and −6.2±1.5 mV in the presence of 30 μM equol, respectively (*P* = NS). Steady-state activation of hKv4.3 was determined using tail current at −40 mV after a brief depolarization step (8 ms) to between −40 and +60 mV from holding potential of −80 mV as described previously[15]. The V1/2 of hKv4.3 activation conductance was positively shifted from 0.7±1.5 mV in control to 9.8±1.7 mV by 30 μM equol (n = 7, *P*<0.01 *vs*. control).

Time-dependent recovery of hKv4.3 current from inactivation was analyzed with a paired-pulse protocol by varying P1–P2 interval as shown in the inset of Fig 3C. The normalized recovery curves were fitted to a mono-exponential function in the absence and presence of equol (Fig 3D). Recovery time constant was 103.4±3.7 ms in control, and 105.6±3.1 ms in the presence of 30 μM equol, respectively (n = 6, *P* = NS). The result suggests that recovery of hKv4.3 current from inactivation is not affected by equol.

### Effects of equol on recombinant cardiac I_Ks_

**Fig 4:**
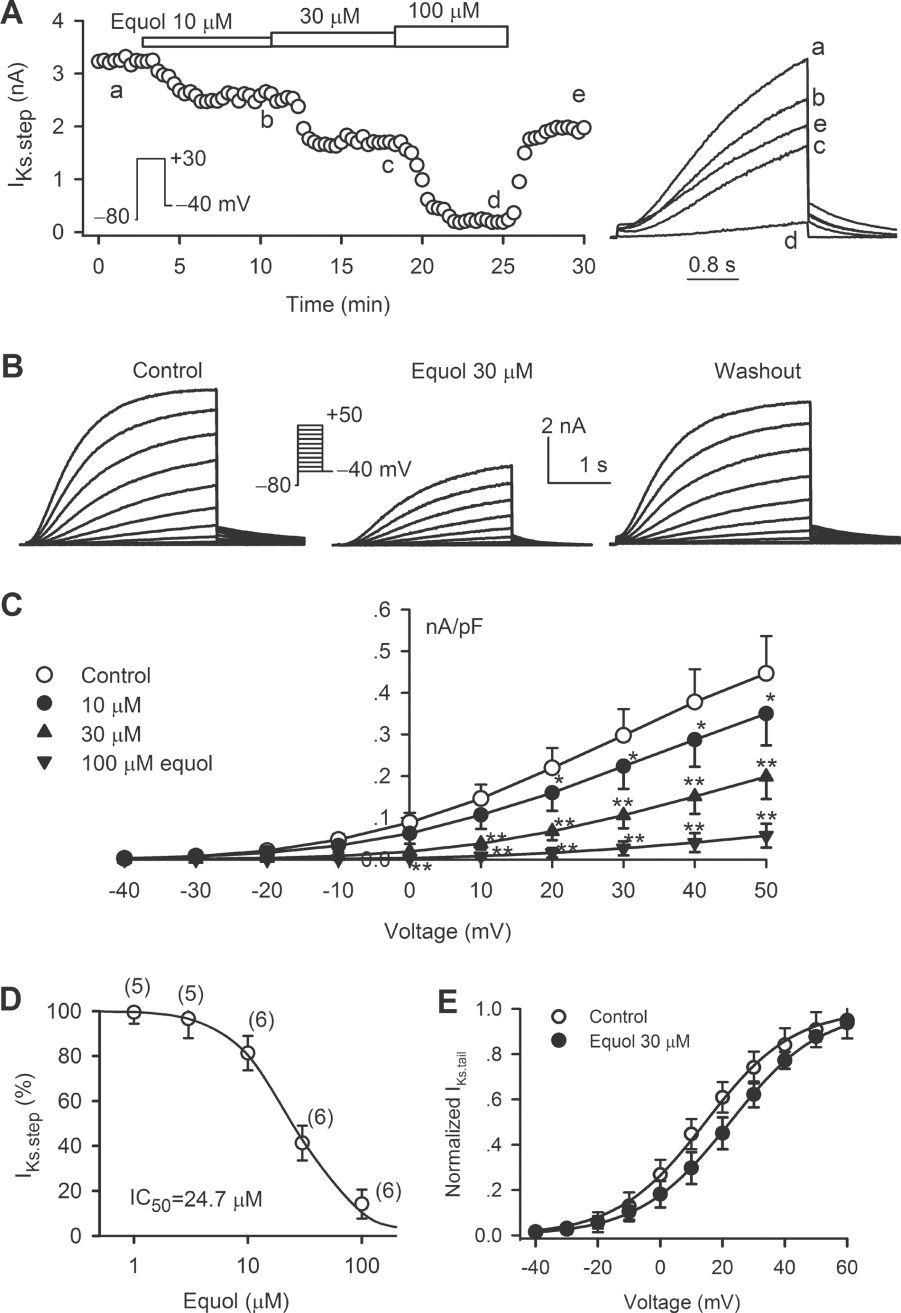
Effect of equol on recombinant *I*_Ks_. (A) Time course of *I*_Ks.step_ recorded in a representative HEK 293 cell stably expressing hKCNQ1/hKCNE1 with the voltage protocol shown in the inset in the absence and presence of 10, 30, and 100 μM equol. Original current traces at corresponding time points are shown in the right. (B) Voltage-dependent *I*_Ks_ traces recorded in a typical experiment with the voltage protocol shown in the inset in the absence and presence of 30 μM equol and upon washout. (C) *I-V* relationships of *I*_Ks.step_ in the absence and presence of 10, 30 and 100 μM equol (n = 6, *P<0.05, **P<0.01 *vs*. control). (D) Concentration-response relationship of *I*_Ks.step_ inhibition by equol at +50 mV was fitted to the Hill equation. The numbers in the parentheses represent experimental number. (E) Variables of normalized *I*_Ks.tail_ were fitted to the Boltzmann equation in the absence and presence of 30 μM equol.

Fig 4A displays the time course of recombinant *I*Ks traces recorded in a representative cell in the absence and presence of 10, 30, and 100 μM equol using a 3-s voltage step to +30 mV every 10 s. Equol inhibited *I*Ks in a concentration-dependent manner. Fig 4B shows the voltage-dependent *I*Ks in another typical experiment with the voltage protocol shown in the inset. Equol at 30 μM substantially suppressed *I*Ks, and the inhibitory effect was reversed by washout. Fig 4C illustrates the *I-V* relationships of *I*Ks.step in the absence and presence of 10, 30 and 100 μM equol. Equol significantly inhibited *I*Ks at test potentials from 0 to +50 mV (n = 6, *P*<0.05, *P*<0.01 *vs*. control). The IC50 of equol for inhibiting *I*Ks.step was 24.7 μM with a Hill coefficient of 1.5 (Fig 4D).

The steady-state activation (g/g_max_) of cardiac *I*_Ks_ was determined using tail current in the absence and the presence of 30 μM equol (Fig 4E). The values were fitted to the Boltzmann function. The V_1/2_ of *I*_Ks_ activation was 14.5±3.1 mV in control and 22.3±3.7 mV in 30 μM equol (n = 6, *P*<0.01 *vs*. control).

### Effect of equol on cardiac hERG channels

**Fig 5:**
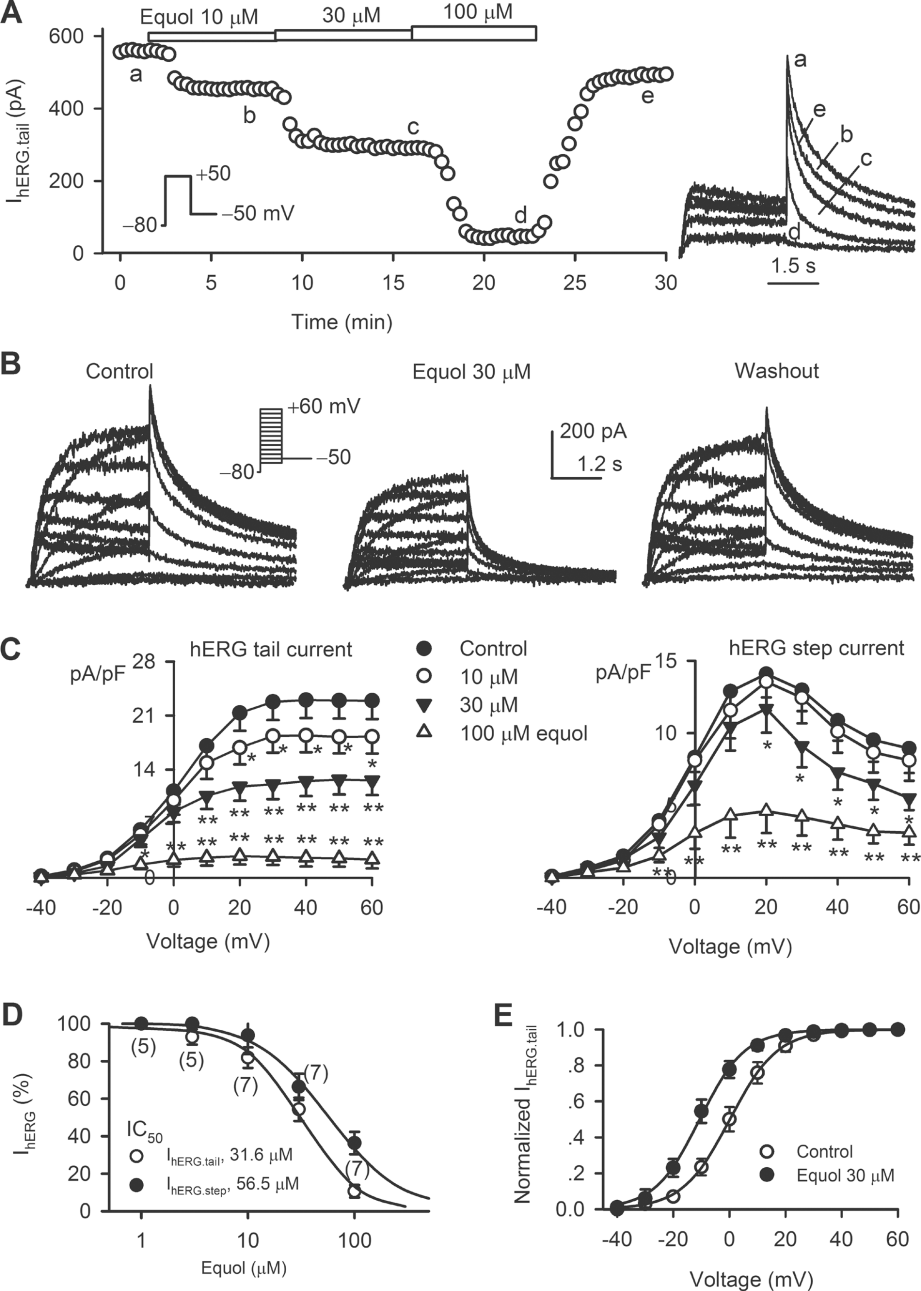
Effects of equol on hERG channels. (A) Time course of *I*_hERG.tail_ recorded in a representative HEK 293 cell stably expressing *KCNH2* with the voltage protocol shown in the inset in the absence and presence of 10, 30, and 100 μM equol. Original current traces at corresponding time points are shown in the right. (B) Voltage-dependent *I*_hERG_ traces recorded in a typical experiment in the absence and presence of 30 μM equol and upon washout. (C) *I-V* relationships of *I*_hERG.tail_ (left) and *I*_hERG.step_ (right) in the absence and presence of 10, 30, and 100 μM equol (n = 7, *P<0.05, **P<0.01 *vs*. control). (D) Concentration-response relationships of equol for inhibiting *I*_hERG.tail_ and I_hERG.step_ were fitted to the Hill equation. The numbers in the parentheses represent experimental number. (E) Variables of normalized *I*_hERG.tail_ in the absence and presence of 30 μM equol were fitted to the Boltzmann equation.

Fig 5A shows the time course of hERG tail current (*I*_hERG.tail_) traces recorded in a HEK 293 cell stably expressing (*KCNH2*) in the absence and presence of 10, 30, and 100 μM equol. Equol decreased *I*_hERG.tail_ in a concentration-dependent manner, and the inhibition was reversed by washout. Similar inhibition and washout were obtained with 30 μM equol for voltage-dependent hERG current (*I*_hERG_) with the voltage protocol shown in the inset (Fig 5B). Equol at 10 μM decreased *I*_hERG.tail_ at +10 mV to +60 mV (n = 6, *P*<0.05, *P*<0.01 *vs*. control), but not *I*_hERG.step_ (Fig 5C). Significant reduction for both *I*_hERG.tail_ and *I*_hERG.step_ was observed at 30 and 100 μM equol (0 mV to +60 mV, n = 7, *P*<0.05, *P*<0.01 *vs*. control). The IC_50_ of equol was 31.6 μM for inhibiting *I*_hERG.tail_, and 56.5 μM for inhibiting *I*_hERG.step_ (Fig 5D).

The steady-state activation (g/g_max_) of *I*_hERG_ was determined by normalizing *I*_hERG.tail_ in control and in the presence of 30 μM equol (Fig 5E). The values were fitted to the Boltzmann function. The V_1/2_ of *I*_hERG_ activation was 0.15±2.5 mV and −10.5±2.8 mV in the absence (control) and presence of 30 μM equol, respectively (n = 6, *P*<0.01 *vs*. control).

### Effect of equol on hKir2.1 current

**Fig 6:**
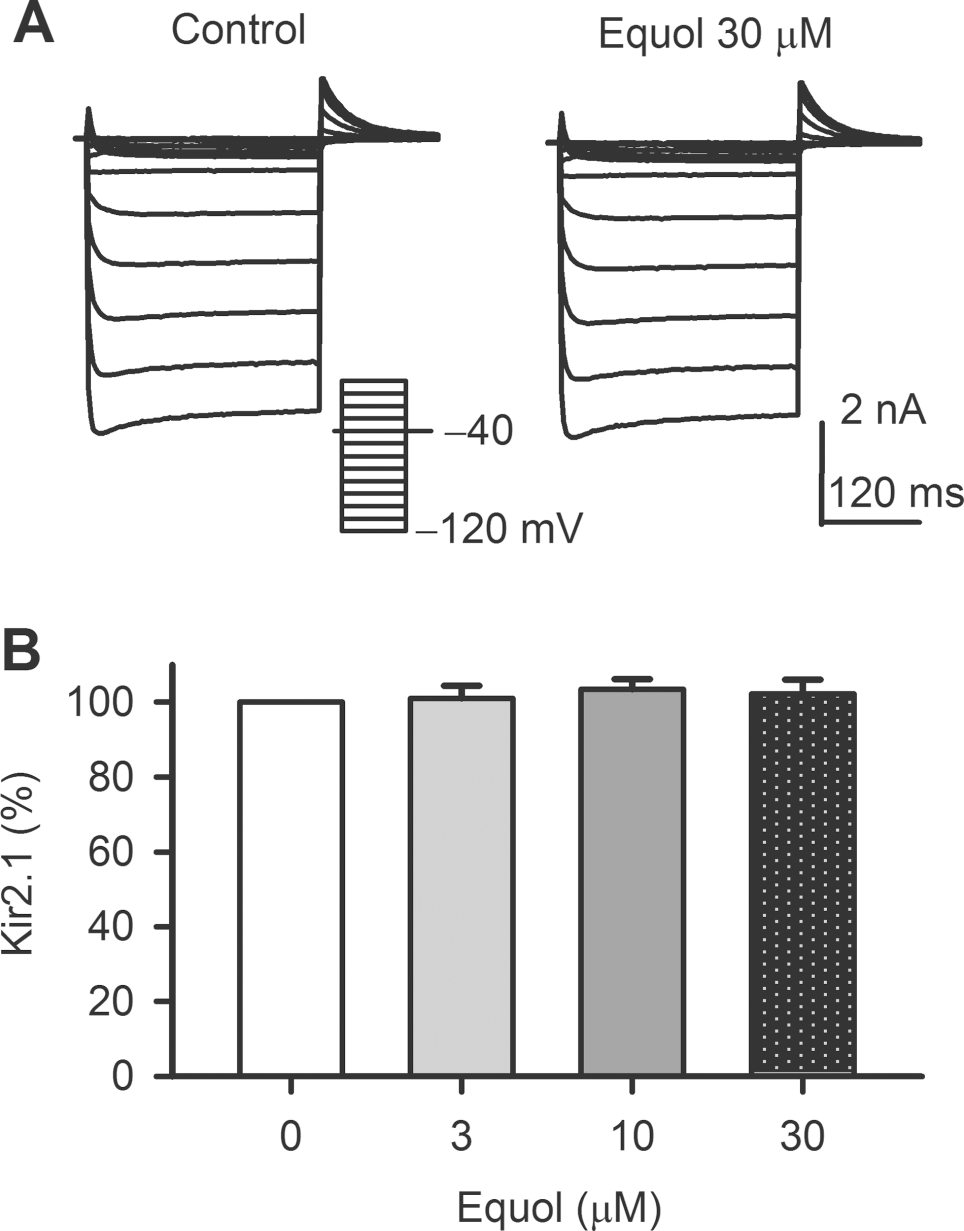
Effect of equol on hKir2.1. (A) Voltage-dependent hKir2.1 current recorded in a representative HEK 293 cell expressing *KCNJ2* in the absence and presence of 30 μM equol. (B) Percentage values of hKir2.1 current at −100 mV in the absence and presence of 3, 10, 30 μM equol (n = 5).

Fig 6A shows the typical hKir2.1 current recorded in a HEK 293 cell expressing *KCNJ2* with the voltage protocol shown in the inset in control and in the presence of 30 μM equol. Equol in the concentration from 3 to 30 μM had no effect on Kir2.1 current (Fig 6B).

### Effect of equol on BK_Ca_ current

**Fig 7:**
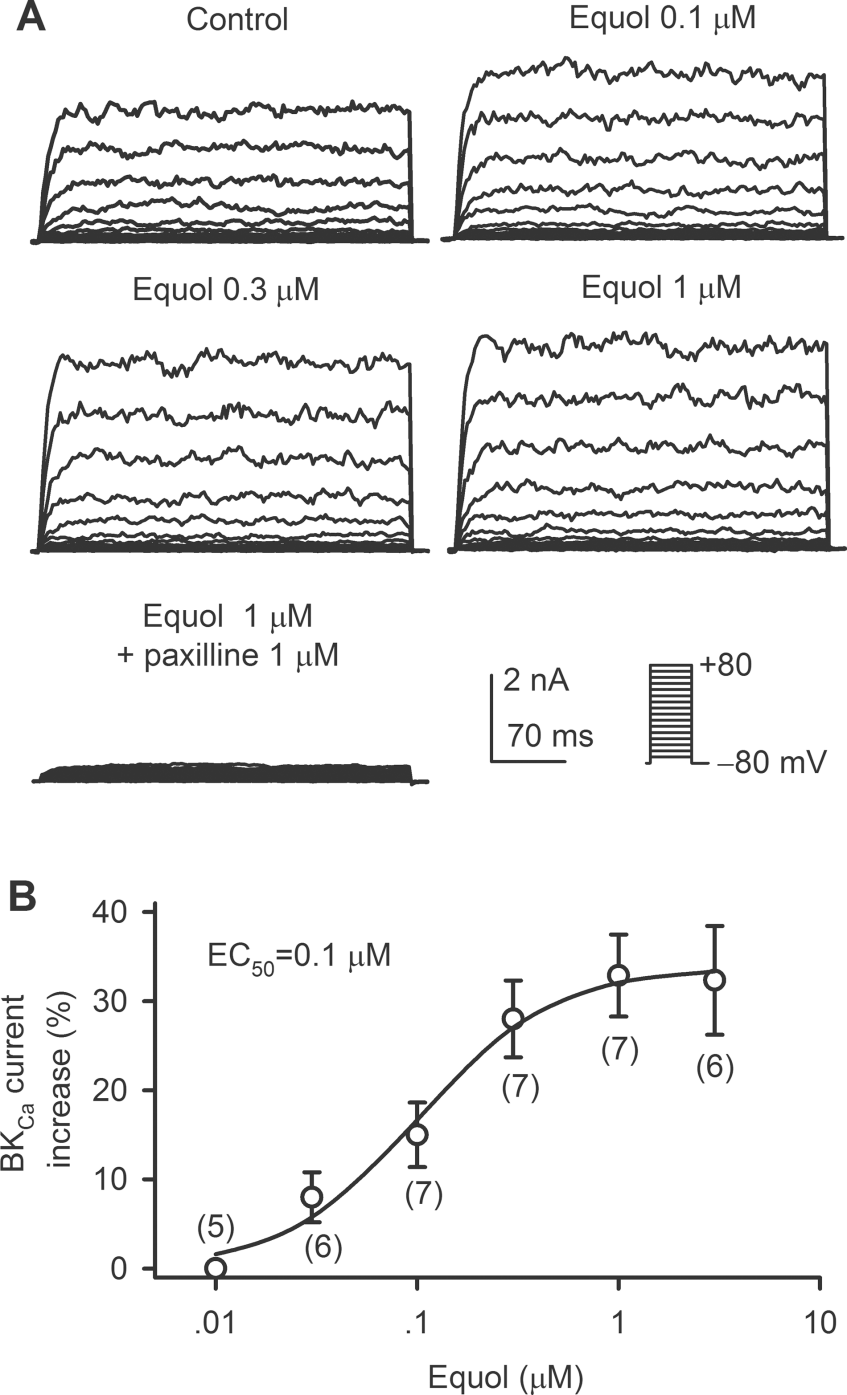
Stimulation of BK_Ca_ channels by equol. (A) Voltage-dependent BK_Ca_ current recorded in a representative HEK 293 cell stably expressing *KCNMA1* and *KCNMB1* with voltage protocol shown in the inset in the absence and presence of 0.1, 0.3 and 1 μM equol, and 1 μM equol plus 1 μM paxilline. (B) Concentration-response curve of equol (0.01−3 μM) for stimulating BK_Ca_ current at +70 mV was fitted to the Hill equation. The numbers in the parentheses represent experimental number.

Our recent study has demonstrated that equol induces vasodilation and increases rat cerebral blood flow by stimulating BKCa channels by acting on its auxiliary β1-subunits[13]. However, the efficacy of equol on BKCa channels was not determined. We therefore determined the EC50 (50% of effective maximum concentration) of equol for increasing BKCa current in HEK 293 cells stably expressing both the core α-subunit (*KCNMA1*) and the auxiliary β1-subunit (*KCNMB1*) of the channel. Fig 7A shows the BKCa current traces recorded in a representative cell with the voltage protocol shown in the inset. BKCa current was increased by 0.1, 0.3 and 1 μM equol. The BKCa channel blocker paxilline (1 μM) almost fully suppressed the current. Fig 7B illustrates the concentration-response curve of equol (0.01–3 μM) for stimulating BKCa current (+70 mV) fitted to the Hill equation. The EC50 of equol for eliciting BKCa current was 0.1 μM with a Hill coefficient of 1.3.

### Effect of equol on ventricular action potential

**Fig 8:**
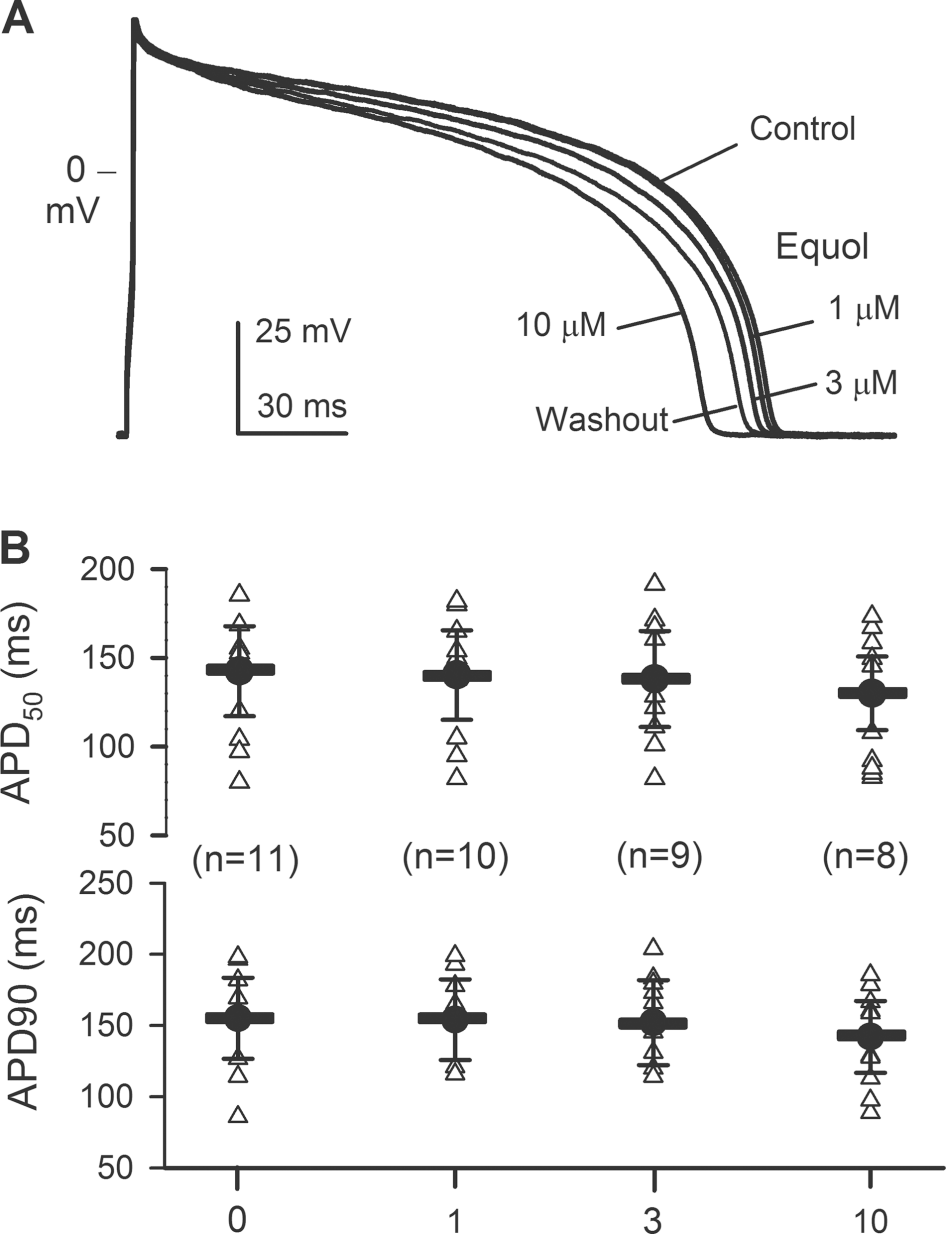
Effects of equol on action potentials in isolated ventricular myocytes from guinea pig hearts. (A) Action potentials recorded (2 Hz) in current clamp mode in a representative myocyte in the absence (control) and presence of 1, 3, and 10 μM equol, and washout. (B) Individual (triangle symbols) and mean (thick horizontal lines) values of APD_50_ and APD_90_ in the absence and presence of 1, 3, and 10 μM equol (n = 8−11, *P* = NS *vs*. control).

Equol at 10 μM significantly inhibited both hERG current and *I*Ks, which implies a potential to cause prolongation of QT interval by increasing ventricular action potential duration, a well-known cardiac toxicity. We therefore determined the effect of equol (1, 3 and 10 μM) on action potential duration (APD) in guinea pig ventricular myocytes (37°C). Fig 8A illustrates the ventricular action potentials recorded (2 Hz) in a representative myocyte in control and in the presence of 1, 3, and 10 μM equol. APD was clearly shortened by 10 μM equol. The effect was partially reversed on drug washout. Fig 8B shows the individual and mean values of APD at 50% and 90% repolarization (APD50 and APD90) in ventricular myocytes of guinea pig hearts. Equol at 1 and 3 μM had no effect on APD50 and APD90. Although 10 μM equol reduced APD50 and APD90 from 141.4±25.4 ms and 155.1±28.4 ms in control to 131.1±20.7 ms and 142.2±25.1 ms, no statistical significance was observed (n = 8, *P*>0.05). Changes in resting membrane potential and action potential amplitude were not observed with equol (1–10 μM, data not shown) treatment, suggesting that equol does not significantly inhibit inward rectifier potassium current (*I*K1).

## Discussion

The present study demonstrates for the first time that equol inhibits cloned hKv1.5, hKv4.3, hKCNQ1/hKCNE1, and hERG channels, while stimulating BK_Ca_ channels, stably expressed in HEK 293 cells. Equol has no effect on hKir2.1 current. The inhibitory effect of equol on K^+^ channels is as follows: hKv4.3>hKv1.5>hKCNQ1/hKCNE1>hERG. Interestingly, the efficacy of equol for stimulating BK_Ca_ channels is 150-fold greater than for inhibiting cardiac K^+^ channels.

Previous studies reported that plasma equol concentrations in human were ranged from ~0 to 130 nM depending upon the type of diet[25]. In a cohort study of healthy adult, serum equol concentration was found to be from 10.3–139 nM (2.5–33.6 mg/L) after consuming 2×250 ml/d soy milk for 3 days[26]. However, little information is available in the literature for plasma protein binding ratio of equol. A recent study reported the plasma levels of free equol and its conjugates in a 24-h period after single oral administration of dietary daidzein (20 mg) in ovariectomized Sprague-Dawley rats using a LC-MS/MS approach[27]. The maximum plasma concentration (Cmax) for total equol (conjugated and unconjugated), unconjugated equol, equol monosulfate and equol glucuronides were 3682±2675 nM, 1.21±0.64 nM, 3.15±3.04 nM and 3349±2799 nM, respectively. This suggests that most of the circulating equol was conjugated-equol, specifically equol glucuronides.

It is well recognized that equol is an active metabolite of daidzein and exists in two enantiomeric forms, R-(+)equol and S-(-)equol. Although both are biologically active, only S-equol is the natural diastereoisomer produced by intestinal bacteria in 20%–30% population in Western countries and 50%–60% in Asian population consuming soy-containing diets[2, 28]. The potential cardiovascular benefits of equol have been widely reported[29–33]; however, information related to ion channel involvement is limited. Only one study reported that equol (40 μM) significantly inhibited L-type Ca2+ current (*I*Ca.L) in guinea pig cardiac ventricular myocytes[34]. Our recent study demonstrated that equol increased cerebral blood flow by vasodilation via activating BKCa channels[13]. The present study provides novel experimental information that equol decreases several cardiovascular K+ channels including hKv1.5, hKv4.3, hKCNQ1/hKCNE1, and hERG channels.

Kv1.5 channels are expressed not only in human atrial myocytes for encoding human atrial *I*Kur responsible for atrial repolarization[35, 36], but also in human pulmonary artery smooth muscle cells in which it plays an important role in regulating membrane potential and vascular tone[37]. *I*Kur/hKv1.5 is considered an atrial-selective target for developing anti-atrial fibrillation drug[38, 39]. The present study demonstrated that equol significantly suppressed hKv1.5 stably expressed in HEK 293 cells (IC50, 15.3 μM). The efficacy of equol for inhibiting hKv1.5 is greater than the parent compound daidzein for inhibiting Kv current in rabbit coronary arterial smooth muscle cells (26% inhibition with 30 μM)[40] and in mouse Schwann cells (no inhibition with 100 μM)[41]. Whether equol can be used to treat atrial fibrillation remains to be studied.

Kv4.3 (*KCND3*) encodes cardiac Ito (transient outward K+ current)[42, 43] or *I*A (voltage-activated A-type K+ current) in smooth muscles or neuronal cells[44]. *I*to is responsible for early rapid repolarization of cardiac action potential in several mammalian species including humans, and the gain-of-function mutation of hKv4.3 is implicated Brugada Syndrome[45]. Equol decreased hKv4.3 current stably expressed in HEK 293 cells with IC50s of 11.9 μM (for current charge area) and 30.3 μM (for peak current), and negatively shifted the inactivation potential and positively shifted activation potential of hKv4.3 current. The efficacy for inhibiting hKv4.3 by equol is greater than daidzein (only 30%–40% inhibition with 300 μM)[46]. Equol, as quinidine for blocking open channel of rat cardiac Ito[22], significantly increased inactivation phase, so that the open channel blockade of equol caused a great reduction of the current charge area at a low concentration range (IC50 = 11.9 μM), which suggests potential effect of treating Brugada Syndrome if plasma concentrations reach 3–10 μM.

It is interesting to note that our recent studies demonstrates that equol provides significant neuroprotection against ischemia/reperfusion injury[33], and increases cerebral blood flow[13] in rat models. Further experiments show that equol dilates rat *ex vivo* cerebral basilar artery by eliciting BKCa channels via acting its auxiliary β1-subunits[13]. The present study showed that the effective concentrations of equol for activating BKCa channels were as low as 0.03 to 3 μM (EC50, 0.1 μM), which is very close to equol’s plasma concentrations. The efficacy was much greater than daidzein (little effect at 1 μM) for stimulating BKCa in vascular smooth cells from rat cerebral basilar arteries[47]. Therefore, equol may be a promising drug candidate for treating patients with brain ischemia.

It is well known that *I*Kr and *I*Ks are encoded respectively by hERG[48] and hKCNQ1/hKCNE1[49], and both are important for repolarization in human heart[35, 50]. The genetic or acquired dysfunction of *I*Kr or *I*Ks may cause cardiac arrhythmias due to prolongation of electrocardiogram (ECG) QT interval induced by delayed ventricular repolarization; therefore, blockade of *I*Kr (hERG) and/or *I*Ks is implicated to be a potential risk of cardiac arrhythmia[51, 52]. In the present study, we found that equol significantly decreased hERG and *I*Ks. However, the concentrations for inhibiting hERG (IC50s, 31.6 μM for *I*hERG.tail and 56.2 μM for *I*hERG.step) and recombinant *I*Ks (IC50, 24.7 μM) are higher than plasma concentrations[25–27]. In addition, equol had no effect on Kir2.1 channels. Interestingly, equol did not prolong ventricular APD at 1 and 3 μM, which are effective concentrations for increasing BKCa current, and decreased cardiac APD at 10 μM, which may be resulted from the L-type Ca2+ current inhibition[34]. Therefore, equol would have little cardiac toxicity potential of prolonging ventricular APD thereby QT interval on ECG.

## Conclusion

Collectively, the present study provides the novel information that equol has a stimulating effect on BK_Ca_ channels at very low concentrations, inhibitory effects on hKv1.5 and hKv4.3 at relatively low concentrations, and inhibitory effects on *I*_Kr_ and *I*_Ks_ at high concentrations. These results suggest that equol may be a promising and safe drug candidate for treating patients with cerebral vascular disorders with limited cardiac toxicity potential.

## References

[1] Atkinson C., Frankenfeld C. L., Lampe J. W. (2005). Gut bacterial metabolism of the soy isoflavone daidzein: exploring the relevance to human health. Experimental Biology and Medicine (Maywood) 230(3), 155–170. 10.1177/153537020523000302 15734719

[2] Setchell K. D. R., Clerici C., Lephart E. D., Cole S. J., Heenan C., Castellani D., et al (2005). S-equol, a potent ligand for estrogen receptor beta, is the exclusive enantiomeric form of the soy isoflavone metabolite produced by human intestinal bacterial flora. The American Journal of Clinical Nutrition 81(5), 1072–1079. 15883431 10.1093/ajcn/81.5.1072

[3] Yuan J. P., Wang J. H., Liu X. (2007). Metabolism of dietary soy isoflavones to equol by human intestinal microflora—implications for health. Molecular Nutrition & Food Research 51(7), 765–781. 10.1002/mnfr.200600262 17579894

[4] Hedlund T. E., Johannes W. U., Miller G. J. (2003). Soy isoflavonoid equol modulates the growth of benign and malignant prostatic epithelial cells in vitro. Prostate 54(1), 68–78. doi: 10.1002/pros.10137 12481257 10.1002/pros.10137

[5] Magee P. J., Allsopp P., Samaletdin A., Rowland I. R. (2014). Daidzein, R-(+)equol and S-(-)equol inhibit the invasion of MDA-MB-231 breast cancer cells potentially via the down-regulation of matrix metalloproteinase-2. European Journal of Nutrition 53(1), 345–350. doi: 10.1007/s00394-013-0520-z 23568763 10.1007/s00394-013-0520-z

[6] Choi E. J., Kim G. H. (2014). The antioxidant activity of daidzein metabolites, Odesmethylangolensin and equol, in HepG2 cells. Molecular Medicine Reports 9(1), 328–332. doi: 10.3892/mmr.2013.1752 24154619 10.3892/mmr.2013.1752

[7] Magee P. J. (2011). Is equol production beneficial to health? The Proceedings of the Nutrition Society 70(1), 10–18. doi: 10.1017/S0029665110003940 21092366 10.1017/S0029665110003940

[8] Mahmoud A. M., Yang W., Bosland M. C. (2014). Soy isoflavones and prostate cancer: a review of molecular mechanisms. The Journal of Steroid Biochemistry and Molecular Biology 140, 116–132. doi: 10.1016/j.jsbmb.2013.12.010 24373791 10.1016/j.jsbmb.2013.12.010 PMC3962012

[9] Rufer C. E., Kulling S. E. (2006). Antioxidant activity of isoflavones and their major metabolites using different in vitro assays. Journal of Agricultural and Food Chemistry 54(8), 2926–2931. doi: 10.1021/jf053112o 16608210 10.1021/jf053112o

[10] Turner R., Baron T., Wolffram S., Minihane A. M., Cassidy A., Rimbach G., et al (2004). Effect of circulating forms of soy isoflavones on the oxidation of low density lipoprotein. Free Radical Research 38(2), 209–216. 15104215 10.1080/10715760310001641854

[11] Jackman K. A., Woodman O. L., Chrissobolis S., Sobey C. G. (2007). Vasorelaxant and antioxidant activity of the isoflavone metabolite equol in carotid and cerebral arteries. Brain Research 1141, 99–107. doi: 10.1016/j.brainres.2007.01.007 17274967 10.1016/j.brainres.2007.01.007

[12] Guo K., Zhang B., Chen C., Uchiyama S., Ueno T., Chen Y., et al (2010). Daidzein-metabolising phenotypes in relation to serum lipids and uric acid in adults in Guangzhou, China. The British Journal of Nutrition 104, 118–124. doi: 10.1017/S0007114510000279 20205965 10.1017/S0007114510000279

[13] Yu W., Wang Y., Song Z., Zhao L. M., Li G. R., Deng X. L. (2016). Equol increases cerebral blood flow in rats via activation of large-conductance Ca(2+)-activated K(+) channels in vascular smooth muscle cells. Pharmacolgical Research 107, 186–194. 10.1016/j.phrs.2016.03.015 26995303

[14] Zhang Y. H., Wu W., Sun H. Y., Deng X. L., Cheng L. C., Li X., et al (2012). Modulation of human cardiac transient outward potassium current by EGFR tyrosine kinase and Src-family kinases. Cardiovascular Research 93(3), 424–433. doi: 10.1093/cvr/cvr347 22198508 10.1093/cvr/cvr347

[15] Wu H. J., Wu W., Sun H. Y., Qin G. W., Wang H. B., Wang P., et al (2011). Acacetin causes a frequency- and use-dependent blockade of hKv1.5 channels by binding to the S6 domain. Journal of Molecular and Cellular Cardiology 51(6), 966–973. doi: 10.1016/j.yjmcc.2011.08.022 21906601 10.1016/j.yjmcc.2011.08.022

[16] Zhang D. Y., Wang Y., Lau C. P., Tse H. F., Li G. R. (2008). Both EGFR kinase and Src-related tyrosine kinases regulate human ether-a-go-go-related gene potassium channels. Cellular Signalling 20(10), 1815–1821. doi: 10.1016/j.cellsig.2008.06.006 18617000 10.1016/j.cellsig.2008.06.006

[17] Dong M. Q., Sun H. Y., Tang Q., Tse H. F., Lau C. P., Li G. R. (2010). Regulation of human cardiac KCNQ1/KCNE1 channel by epidermal growth factor receptor kinase. Biochimica Biophysica Acta 1798(5), 995–1001. 10.1016/j.bbamem.2010.01.010 20085748

[18] Zhang D. Y., Wu W., Deng X. L., Lau C. P., Li G. R. (2011). Genistein and tyrphostin AG556 inhibit inwardly-rectifying Kir2.1 channels expressed in HEK 293 cells via protein tyrosine kinase inhibition. Biochimica Biophysica Acta 1808(8), 1993–1999. 10.1016/j.bbamem.2011.04.015 21570948

[19] Wu W., Wang Y., Deng X. L., Sun H. Y., Li G. R. (2013). Cholesterol down-regulates BK channels stably expressed in HEK 293 cells. PLoS One 8, e79952 doi: 10.1371/journal.pone.0079952 24260325 10.1371/journal.pone.0079952 PMC3832390

[20] Li G. R., Lau C. P., Shrier A. (2002). Heterogeneity of sodium current in atrial vs epicardial ventricular myocytes of adult guinea pig hearts. Journal of Molecular and Cellular Cardiology 34(9), 1185–1194. 12392892 10.1006/jmcc.2002.2053

[21] Liu H., Sun H. Y., Lau C. P., Li G. R. (2007). Regulation of voltage-gated cardiac sodium current by epidermal growth factor receptor kinase in guinea pig ventricular myocytes. Journal of Molecular and Cellular Cardiology 42 (4), 760–768. doi: 10.1016/j.yjmcc.2006.10.013 17188293 10.1016/j.yjmcc.2006.10.013

[22] Clark R. B., Sanchez-Chapula J, Salinas-Stefanon E, Duff H. J., Giles W. R. (1995). Quinidine-induced open channel block of K^+^ current in rat ventricle. British Journal of Pharmacology 115(2), 335–343. 7670736 10.1111/j.1476-5381.1995.tb15882.x PMC1908313

[23] Oyama Y., Harata N, Akaike N (1992). Accelerating action of quinidine on the decay phase of transient outward current in dissociated hippocampal pyramidal neurons of rats. The Japanese Journal of Pharmacology 58(2), 185–188 1507524 10.1254/jjp.58.185

[24] Yang L., Liu H., Sun H. Y., Li G. R. (2015). Intravenous anesthetic propofol inhibits multiple human cardiac potassium channels. Anesthesiology 122 (3), 571–584. doi: 10.1097/ALN.0000000000000495 25321870 10.1097/ALN.0000000000000495

[25] Blair R. M., Appt S. E., Franke A. A., Clarkson T. B. (2003). Treatment with antibiotics reduces plasma equol concentration in cynomolgus monkeys (Macaca fascicularis). The Journal of Nutrition 133(7), 2262–2267. 12840190 10.1093/jn/133.7.2262

[26] Setchell K. D. R., Cole S. J. (2006). Method of defining equol-producer status and its frequency among vegetarians. The Journal of Nutrition 136(8), 2188–2193. 16857839 10.1093/jn/136.8.2188

[27] Legette L. L., Prasain J., King J., Arabshahi A., Barnes S., Weaver C. M. (2014). Journal of Agriculture and Food Chemistry 62(6), 1294–1300. 10.1021/jf400097m PMC3983397 24446705

[28] Setchell K. D. R., Clerici C. (2010). Equol: pharmacokinetics and biological actions. The Journal of Nutrition 140(7), 1363S–1368S. doi: 10.3945/jn.109.119784 20519411 10.3945/jn.109.119784 PMC2884334

[29] Jackman K. A., Woodman O. L., Sobey C. G. (2007). Isoflavones, equol and cardiovascular disease: pharmacological and therapeutic insights. Current Medicinal Chemistry 14(26), 2824–2830. 18045128 10.2174/092986707782360178

[30] Rowlands D. J., Chapple S., Siow R. C., Mann G. E. (2011). Equol-stimulated mitochondrial reactive oxygen species activate endothelial nitric oxide synthase and redox signaling in endothelial cells: roles for F-actin and GPR30. Hypertension 57(4), 833–840. doi: 10.1161/HYPERTENSIONAHA.110.162198 21300668 10.1161/HYPERTENSIONAHA.110.162198 PMC3086276

[31] Hazim S., Curtis P. J., Schar M. Y., Ostertag L. M., Kay C. D., Minihane A. M., et al (2016). Acute benefits of the microbial-derived isoflavone metabolite equol on arterial stiffness in men prospectively recruited according to equol producer phenotype: a double-blind randomized controlled trial. The American Journal of Clinical Nutrition 103(3), 694–702. doi: 10.3945/ajcn.115.125690 26843154 10.3945/ajcn.115.125690 PMC4763500

[32] Ohkura Y., Obayashi S., Yamada K., Yamada M., Kubota T. (2015). S-equol partially restored endothelial nitric oxide production in isoflavone-deficient ovariectomized rats. Journal of Cardiovascular Pharmacology 65(5), 500–507. doi: 10.1097/FJC.0000000000000220 25636070 10.1097/FJC.0000000000000220

[33] Yu W., Wang Y., Zhou D. X., Zhao L. M., Li G. R., Deng X. L. (2014). Equol is neuroprotective during focal cerebral ischemia and reperfusion that involves p-Src and gp91(phox). Current Neurovascular Research 11(4), 367–377. 25198009 10.2174/1567202611666140908094517

[34] Liew R., Williams J. K., Collins P., MacLeod K. T. (2003). Soy-derived isoflavones exert opposing actions on guinea pig ventricular myocytes. The Journal of Pharmacology and Experimental Therapeutics 304(3), 985–993. doi: 10.1124/jpet.102.042986 12604673 10.1124/jpet.102.042986

[35] Li G. R., Feng J., Yue L., Carrier M., Nattel S. (1996). Evidence for two components of delayed rectifier K^+^ current in human ventricular myocytes. Circulation Research 78(4), 689–696. 8635226 10.1161/01.res.78.4.689

[36] Feng J., Wible B., Li G. R., Wang Z., Nattel S. (1997). Antisense oligodeoxynucleotides directed against Kv1.5 mRNA specifically inhibit ultrarapid delayed rectifier K^+^ current in cultured adult human atrial myocytes. Circulation Research 80(4), 572–579. 9118489 10.1161/01.res.80.4.572

[37] Remillard C. V., Tigno D. D., Platoshyn O., Burg E. D., Brevnova E. E., Conger D., et al (2007). Function of Kv1.5 channels and genetic variations of KCNA5 in patients with idiopathic pulmonary arterial hypertension. American Journal of Physiology-Cell Physiology 292(5), C1837–1853. doi: 10.1152/ajpcell.00405.2006 17267549 10.1152/ajpcell.00405.2006

[38] Li G. R., Sun H. Y., Zhang X. H., Cheng L. C., Chiu S. W., Tse H. F., et al (2009). Omega-3 polyunsaturated fatty acids inhibit transient outward and ultra-rapid delayed rectifier K^+^currents and Na^+^current in human atrial myocytes. Cardiovascular Research 81(2), 286–293. doi: 10.1093/cvr/cvn322 19029136 10.1093/cvr/cvn322

[39] Li G. R., Wang H. B., Qin G. W., Jin M. W., Tang Q., Sun H. Y., et al (2008). Acacetin, a natural flavone, selectively inhibits human atrial repolarization potassium currents and prevents atrial fibrillation in dogs. Circulation 117(19), 2449–2457. doi: 10.1161/CIRCULATIONAHA.108.769554 18458165 10.1161/CIRCULATIONAHA.108.769554

[40] Ko E. A., Park W. S., Son Y. K., Kim D. H., Kim N., Kim H. K., et al (2009). The effect of tyrosine kinase inhibitor genistein on voltage-dependent K^+^ channels in rabbit coronary arterial smooth muscle cells. Vascular Pharmacology 50(1), 51–56. 18952004 10.1016/j.vph.2008.09.004

[41] Peretz A., Sobko A., Attali B. (1999). Tyrosine kinases modulate K^+^ channel gating in mouse Schwann cells. The Journal of Physiology 519(Pt 2), 373–384. 10457056 10.1111/j.1469-7793.1999.0373m.x PMC2269503

[42] Li G. R., Dong M. Q. (2010). Pharmacology of cardiac potassium channels. Advances in Pharmacology 59, 93–134. doi: 10.1016/S1054-3589(10)59004-5 20933200 10.1016/S1054-3589(10)59004-5

[43] Dixon J. E., Shi W., Wang H. S., McDonald C., Yu H., Wymore R. S., et al (1996). Role of the Kv4.3 K^+^ channel in ventricular muscle. A molecular correlate for the transient outward current. Circulation Research 79(4), 659–668. 8831489 10.1161/01.res.79.4.659

[44] Amberg G. C., Koh S. D., Imaizumi Y., Ohya S., Sanders K. M. (2003). A-type potassium currents in smooth muscle. American Journal of Physiology-Cell Physiology 284(3), C583–595. doi: 10.1152/ajpcell.00301.2002 12556357 10.1152/ajpcell.00301.2002

[45] Giudicessi J. R., Ye D., Tester D. J., Crotti L., Mugione A., Nesterenko V. V., et al (2011). Transient outward current (I(to)) gain-of-function mutations in the KCND3-encoded Kv4.3 potassium channel and Brugada syndrome. Heart Rhythm 8(7), 1024–1032. doi: 10.1016/j.hrthm.2011.02.021 21349352 10.1016/j.hrthm.2011.02.021 PMC3150551

[46] Kim H. J., Ahn H. S., Choi B. H., Hahn S. J. (2011). Inhibition of Kv4.3 by genistein via a tyrosine phosphorylation-independent mechanism. American Journal of Physiology-Cell Physiology 300(3), C567–575. doi: 10.1152/ajpcell.00031.2010 21148405 10.1152/ajpcell.00031.2010 PMC3063965

[47] Zhang H. T., Wang Y., Deng X. L., Dong M. Q., Zhao L. M., Wang Y. W. (2010). Daidzein relaxes rat cerebral basilar artery via activation of large-conductance Ca^2+^-activated K^+^ channels in vascular smooth muscle cells. European Journal of Pharmacology 630(1–3), 100–106. doi: 10.1016/j.ejphar.2009.12.032 20044987 10.1016/j.ejphar.2009.12.032

[48] Sanguinetti M. C., Jiang C., Curran M. E., Keating M. T. (1995). A mechanistic link between an inherited and an acquired cardiac arrhythmia: HERG encodes the I_Kr_ potassium channel. Cell 81(2), 299–307. 7736582 10.1016/0092-8674(95)90340-2

[49] Sanguinetti M. C., Curran M. E., Zou A., Shen J., Spector P. S., Atkinson D. L., et al (1996). Coassembly of K(V)LQT1 and minK (IsK) proteins to form cardiac I(Ks) potassium channel. Nature 384(6604), 80–83. doi: 10.1038/384080a0 8900283 10.1038/384080a0

[50] Li G. R., Lau C. P., Leung T. K., Nattel S. (2004). Ionic current abnormalities associated with prolonged action potentials in cardiomyocytes from diseased human right ventricles. Heart Rhythm 1(4), 460–468. doi: 10.1016/j.hrthm.2004.06.003 15851200 10.1016/j.hrthm.2004.06.003

[51] Farkas A.S., Nattel S. (2010). Minimizing repolarization-related proarrhythmic risk in drug development and clinical practice. Drugs 70(5), 573–603. doi: 10.2165/11535230-000000000-00000 20329805 10.2165/11535230-000000000-00000

[52] Veerman C. C., Verkerk A. O., Blom M. T., Klemens C. A., Langendijk P. N., van Ginneken A. C., et al (2013). Slow delayed rectifier potassium current blockade contributes importantly to drug-induced long QT syndrome. Circulation Arrhythmia and Electrophysiology 6(5), 1002–1009. doi: 10.1161/CIRCEP.113.000239 23995305 10.1161/CIRCEP.113.000239

